# Performance of Deep Learning Architectures and Transfer Learning for Detecting Glaucomatous Optic Neuropathy in Fundus Photographs

**DOI:** 10.1038/s41598-018-35044-9

**Published:** 2018-11-12

**Authors:** Mark Christopher, Akram Belghith, Christopher Bowd, James A. Proudfoot, Michael H. Goldbaum, Robert N. Weinreb, Christopher A. Girkin, Jeffrey M. Liebmann, Linda M. Zangwill

**Affiliations:** 1 Hamilton Glaucoma Center, Shiley Eye Institute, Department of Ophthalmology, UC San Diego, La Jolla, CA United States; 2 School of Medicine, University of Alabama-Birmingham, Birmingham, AL United States; 3 Bernard and Shirlee Brown Glaucoma Research Laboratory, Edward S. Harkness Eye Institute, Department of Ophthalmology, Columbia University Medical Center, New York, NY United States

## Abstract

The ability of deep learning architectures to identify glaucomatous optic neuropathy (GON) in fundus photographs was evaluated. A large database of fundus photographs (n = 14,822) from a racially and ethnically diverse group of individuals (over 33% of African descent) was evaluated by expert reviewers and classified as GON or healthy. Several deep learning architectures and the impact of transfer learning were evaluated. The best performing model achieved an overall area under receiver operating characteristic (AUC) of 0.91 in distinguishing GON eyes from healthy eyes. It also achieved an AUC of 0.97 for identifying GON eyes with moderate-to-severe functional loss and 0.89 for GON eyes with mild functional loss. A sensitivity of 88% at a set 95% specificity was achieved in detecting moderate-to-severe GON. In all cases, transfer improved performance and reduced training time. Model visualizations indicate that these deep learning models relied on, in part, anatomical features in the inferior and superior regions of the optic disc, areas commonly used by clinicians to diagnose GON. The results suggest that deep learning-based assessment of fundus images could be useful in clinical decision support systems and in the automation of large-scale glaucoma detection and screening programs.

## Introduction

Glaucoma is characterized by progressive structural and functional damage to the optic nerve head that can eventually lead to functional impairment, disability, and blindness. It is one of the most common causes of irreversible blindness worldwide and is expected to affect roughly 80 million people by 2020^1–3^. Current estimates suggest that roughly 50% of people suffering from glaucoma in the developed world are currently undiagnosed and aging populations suggests that the impact of glaucoma will continue to rise^4,5^. Effective screening programs to detect glaucoma are needed to address this growing public health problem^6,7^. Despite extensive research on glaucoma screening, the U.S. Preventive Services Task Force does not recommend population screening for glaucoma at least in part due the relatively low sensitivity and specificity of glaucoma screening tests given the low prevalence of the disease and insufficient evidence that the benefits of screening outweigh the costs and potential harm^8^.

Traditionally, clinicians have examined the ONH with ophthalmoscopy and fundus photography to diagnose and monitor glaucoma. Subjective qualitative ONH evaluation requires significant clinical training and agreement is limited even among subspecialty-trained clinicians^9–11^. Automated methods that use techniques from artificial intelligence to objectively interpret images of the ONH and surrounding fundus can help address this issue. These automated methods may also be used as part of decision support systems in clinical management of glaucoma through incorporation into fundus cameras, electronic medical record systems, or picture archiving and communication systems (PACS). If these automated systems can sufficiently improve sensitivity and specificity beyond previously studied glaucoma-associated measurements, they may pave the way for large-scale screening programs. The Food and Drug Administration (FDA) has recently approved the use of an automated system to review fundus images for diabetic retinopathy^12^. Similar programs for glaucoma may help improve detection rates and identify patients in need of referral to glaucoma specialists.

Over the past several years, deep learning techniques have advanced the state-of-the-art in image classification, segmentation, and object detection in medical and ophthalmic images^13–15^. In particular, deep convolutional neural networks (CNN) have found use in ophthalmology for tasks that include identifying diabetic retinopathy in fundus images, interpreting and segmenting optical coherence tomography (OCT) images, and detecting drusen, neovascularization, and macular edema by OCT^16–19^. As opposed to traditional machine learning approaches that rely on features explicitly defined by experts using their domain knowledge^20–23^, CNNs are a type network that can learn features to maximize the network’s ability to distinguish between healthy and disease images. There are many different CNN architectures that have been designed to perform image classification and segmentation tasks^24^. Each of these architectures differ in specific aspects including the number and size of features, the connections between these features, and the overall network depth. Because different architectures are best suited for different problems and it is difficult to know *a priori* what architecture is the right choice for a given task, empirical testing is often the best way to make this determination^25^. A problem common to all deep learning networks, however, is that they require a much larger amount of data and computation time compared to more traditional machine learning techniques. In order to reduce these requirements, transfer learning has become an important technique to apply features learned to perform one task to be applied to other tasks^26^. While there are very large (millions of images) datasets available for training general image classifiers, this scale of data is unavailable in most medical image classification tasks. By using transfer learning, networks trained on general image datasets can be used as a starting point for an ophthalmic task (e.g. classifying fundus images of the ONH)^26^. Transfer learning has previously been employed to train models to detect glaucoma and a variety of other diseases from OCT images^18,27^.

The aim of this work is to develop and evaluate deep learning approaches to identify glaucomatous damage in ONH fundus images. Using a large database of ONH-centered fundus images, we evaluated several different CNN architectures based on glaucoma diagnostic accuracy. Additionally, we quantitatively evaluated the effect of using transfer learning in training these networks.

## Methods

### Data Collection

Study participants were selected from two prospective longitudinal studies designed to evaluate optic nerve structure and visual function in glaucoma: The African Descent and Glaucoma Evaluation Study (ADAGES clinicaltrials.gov identifier: NCT00221923) and the University of California, San Diego (UCSD) based Diagnostic Innovations in Glaucoma Study (DIGS, clinicaltrials.gov identifier: NCT00221897)^28^. The ADAGES is a multicenter collaboration of the UCSD Hamilton Glaucoma Center, Columbia University Medical Center Edward S. Harkness Eye Institute (New York, NY), and the University of Alabama at Birmingham Department of Ophthalmology (Birmingham, AL). These are ongoing, longitudinal studies that included periodic collection of stereo fundus images. Recruitment and methodology was approved by each institution’s Institutional Review Board which adhered to the Declaration of Helsinki and the Health Insurance Portability and Accountability Act. Informed consent was obtained from all participants at recruitment. Table 1 summarizes this dataset.

**Table 1:** Clinical and demographic characteristics of the optic nerve head (ONH) photograph dataset.

Parameter	Healthy	GON
Number of participants	1561	768
Number of eyes	2920	1443
Number of images	9189	5633
Visual Field Mean Deviation (dB)	−0.76 ± 2.1	−4.1 ± 6.0
Age (years)	53.3 ± 14.4	64.0 ± 12.9
Sex (% female)	63.0	57.0
Race (%)		
White/European descent	64.2	50.8
Black/African descent	28.0	45.5
Asian descent	5.3	2.1
Other/Unreported	2.5	1.6

All fundus images included in this work were captured on film as simultaneous stereoscopic ONH photographs using the Nidek Stereo Camera Model 3-DX (Nidek Inc, Palo Alto, California). Film stereophotographs were reviewed by two independent, masked graders for the presence of glaucomatous optic neuropathy (GON) using a stereoscopic viewer. If these graders disagreed, a third experienced grader adjudicated. For analysis, photographs were digitized by scanning 35 mm film slides and storing them as high resolution (~2200 × ~1500 pixels) TIFF images. Stereo image pairs were split into individual images of the ONH for analysis. The total dataset consisted of 7,411 stereo pairs split into 14,822 individual ONH images collected from 4,363 eyes of 2,329 individuals. In this analysis, the data was considered cross-sectionally with a binary class (healthy vs. GON) assigned at the image level.

Standard automated perimetry visual field (VF) testing using a Humphrey Field Analyzer II (Carl Zeiss Meditec, Dubin, CA) with a standard 24-2 testing pattern and the Swedish Interactive Thresholding Algorithm was performed on a semi-annual basis. VF tests with more than 33% fixation losses, 33% false negative errors, or 15% false positive errors were excluded. For each ONH image, the mean deviation (MD) from VF testing closest in time to image acquisition (up to a maximum of one year) was computed and used to estimate VF function at the time of imaging. Visual fields were processed and evaluated for quality according to standard protocols by the UCSD Visual Field Assessment Center^29^.

### Preprocessing and Data Augmentation

Prior to training the deep learning models, a region centered on the ONH was automatically extracted from each fundus image. For our analysis, a separate deep learning model was built on a subset of the training data to estimate the location of the disc center. To provide ground truth for this model, a single human reviewer marked the ONH center in each of the examples. The resulting model was applied to the entire dataset to estimate the ONH center in each image. A square region surrounding the estimated center was the extracted from each image for use in the analysis. Each cropped image was manually reviewed to ensure that each was correctly centered on the ONH.

**Figure 1:**
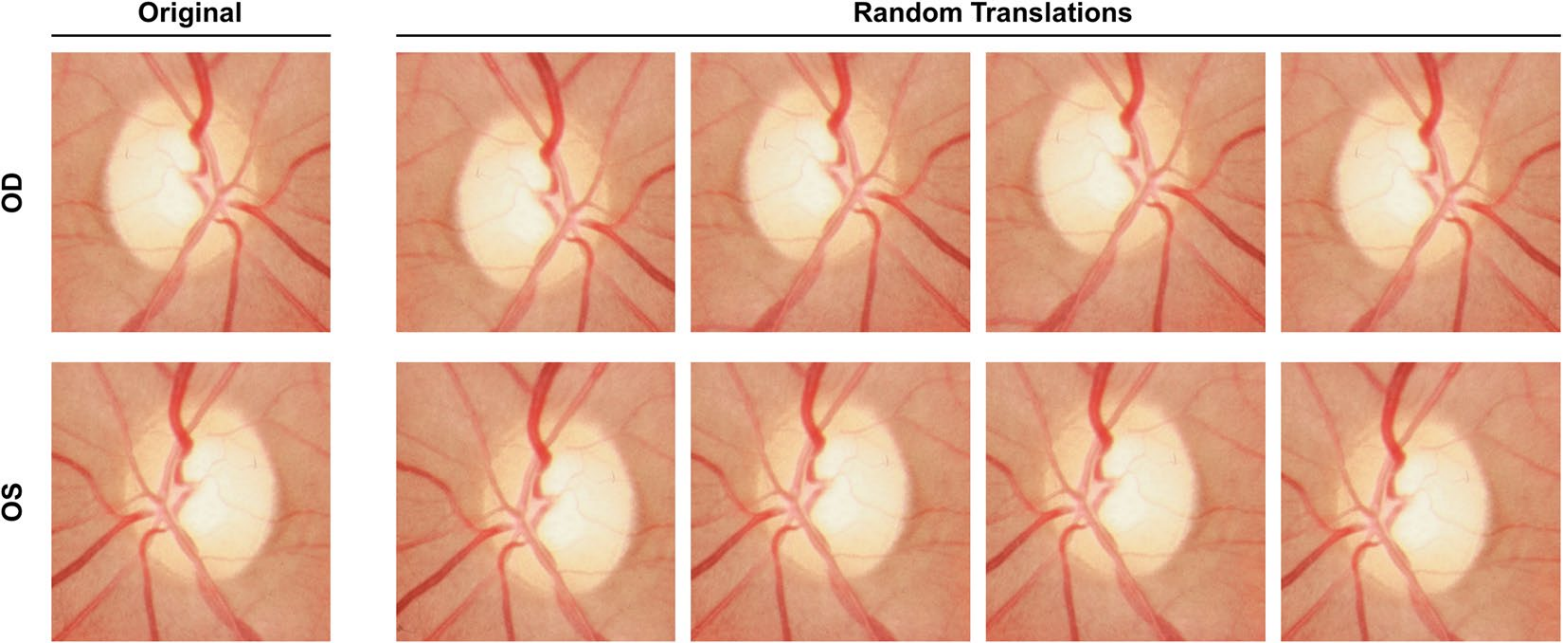
Example input ONH image (top left) and images resulting from data augmentation. Each input image was augmented by horizontal mirroring to mimic alternative OD/OS orientation and random cropping to produce translations of the image.

A data augmentation procedure was also applied to increase the amount and type of variation within the training data. Data augmentation is commonly used in image classification tasks and can result in better performing, more generalizable models that are invariant to certain types of image transformations and variations in image quality^30^. First, horizontally mirrored versions of all images were added to the dataset to mimic the inclusion of both OD and OS orientations of each image. Next, a random cropping procedure was applied to all images (original and flipped). Specifically, the center of the ONH region cropped from each image was randomly perturbed by a small amount to help make the trained models invariant to translational changes and reflect clinical care in which ONH photographs are not always well-centered. This random cropping was repeated five times for each image. The total augmented dataset contained 148,220 images resulting from (14,822 images) × (2 orientations) × (5 random crops). Each augmented image was assigned the same label (healthy or GON) as the original input image from which it was derived. Figure 1 shows an example input image and the corresponding augmentations. After augmentation, all ONH images were down-sampled to a common size of 224 × 224 pixels.

### Deep Learning Models

**Figure 2:**
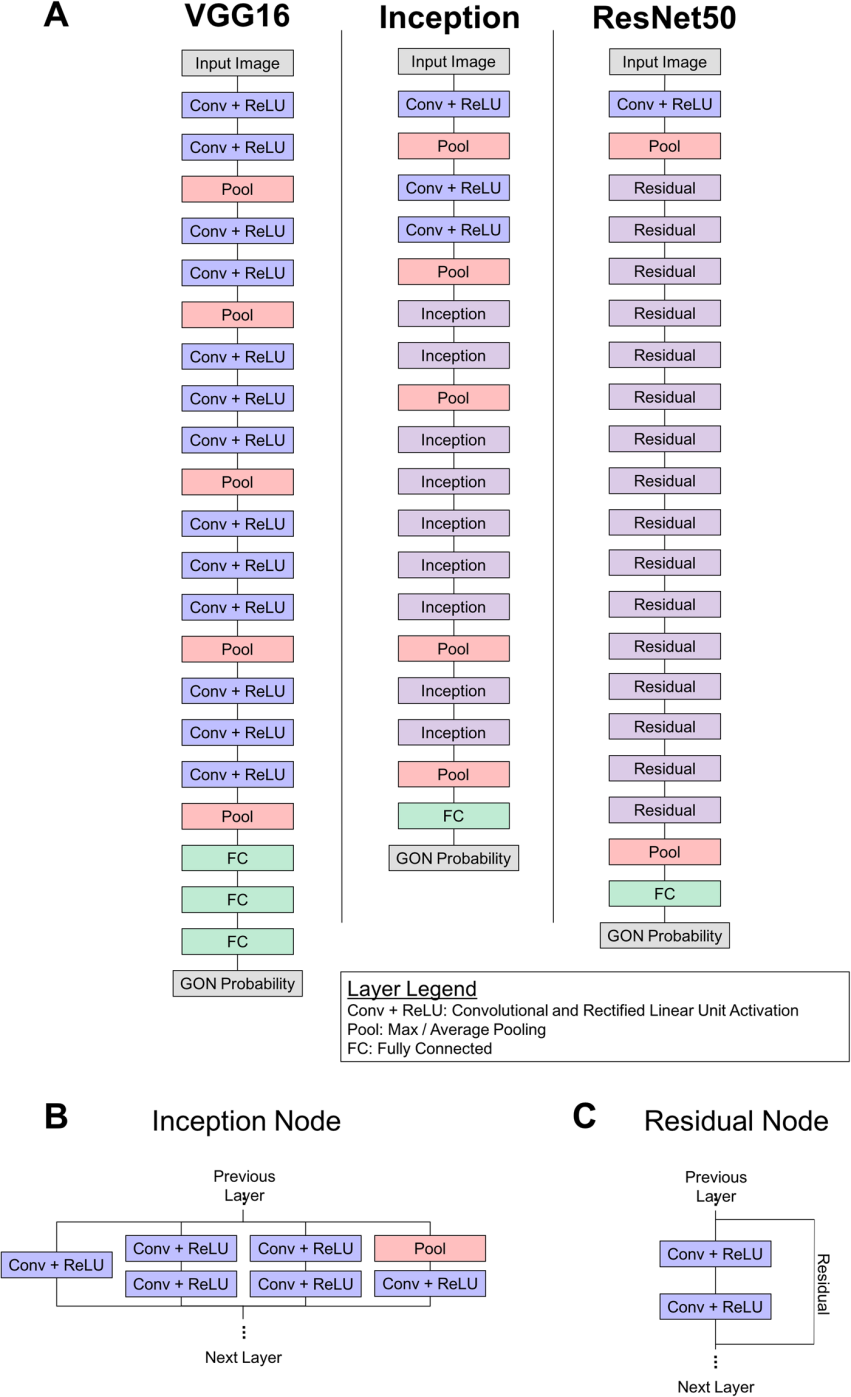
(**A**) Schematics of the three CNN architectures evaluated here. These architectures included VGG16, Inception, and ResNet50. The Inception (**B**) and Residual (**C**) are used as building blocks for the Inception and ResNet50 architectures, respectively.

Three different deep learning architectures were evaluated: VGG16^31^, Inception v3^32^, and ResNet50^33^. These architectures were chosen because they have been widely adopted for both general and medical image classification tasks and their performances are commonly used as a comparison for other architectures^34^. Broadly, these architectures are all similar in that they consist of convolutional and pooling layers arranged in sequential units and a final, fully connected layer that produces an image-level classification. Each architecture differs in the specific arrangement and number of these layers (Fig. 2). Additionally, versions of these models that have been trained on a large, general dataset are available so that a transfer learning approach can be adopted.

For each architecture under consideration, two different versions were evaluated: native and transfer learning. In the native version, model weights were randomly initialized^35^ and training was performed using only the fundus ONH data described here. In the transfer learning version, model weights were initialized based on pretraining on a general image dataset, except for the final, fully connected layers which were randomly initialized. Additional training was performed using fundus images to fine-tune these networks for distinguishing healthy vs. GON images. In all transfer learning cases, models were initially trained using the ImageNet database^36^. ImageNet is an image dataset containing thousands of different objects and scenes used to train and evaluate image classification models.

### Model Training and Selection

The image dataset was divided randomly divided into multiple folds so that 10-fold cross validation could be performed to estimate model performance. Within each fold, the dataset was partitioned into independent training, validation, and testing sets using an 85-5-10 percentage split by participant. This separation was performed at the participant (not image) level. This means that all images from a participant were included in the same partition (training, validation, or testing). This ensured that testing set contained only images of novel eyes that had not been encountered by the model during training. For training, a total of 50,000 iterations with batch size of 25 (~10 epochs) were performed. Based on validation set performance, this was well past the point of convergence. After every 1,000 training iterations, the model was applied to the validation dataset to evaluate performance on unseen data. The model that achieved the highest performance on the validation dataset was selected for evaluation on the testing dataset. This process was repeated for each architecture (VGG16, Inception, ResNet), approach (native, pretrained), and cross validation fold. Table 2 summarizes training data and parameters.

**Table 2:** Summary of the parameters used in training the deep learning models.

Parameter	Value
Weight Initialization	Native: Random initialization, Transfer learning: Pre-trained on ImageNet
Training set sample size/fold	2,006 participants, ~3,710 eyes, ~12,595 images
Validation set sample size/fold	97 participants, ~218 eyes, ~745 images
Testing set sample size/fold	226 participants, ~436 eyes, ~1,482 images
Input image size	224 × 224 pixels
Network output	Softmax probability of GON
Number of training iterations	50,000 (~10 epochs)
Learning rate	0.005 (iters. 0–100,00)

All models were trained and evaluated on a machine running CentOS 6.6 using an NVIDIA Tesla K80 graphics card. Model specification, training and optimization, and application were performed using Caffe tools^37^.

### Model Evaluation

In each cross validation fold, evaluation was performed on the independent test dataset. Native and transfer learning versions of each architecture were evaluated using area under the receiver operating characteristic curve (ROC, AUC). Each model was applied to the augmented test images to generate a probability of GON ranging from 0.0 (predicted healthy) to 1.0 (predicted GON). The probabilities of each augmented image derived from the same original input image were averaged to generate a final image-level prediction.

Using the expert adjudicated grades as the ground truth, the mean and standard deviation of AUC values for each model was estimated across cross validation folds. AUCs were compared across models to identify statistically significant (p < 0.05) differences. Because the testing set contained multiple images of the same participant, considering the classification of each image independently may skew AUC estimates. To address this issue, a clustered AUC approach was used^38^. This approach using random resampling of the data to estimate AUC in the presence of multiple results from the same participant. Statistical analyses were performed using statistical packages in R^39–41^.

After identifying the best performing model, we evaluated its performance in separating healthy eyes from those with mild functional loss and those with moderate or severe loss. To perform this evaluation, GON images in the testing set were stratified by degree of functional loss into two groups: mild and moderate-to-severe. Images graded as GON by the reviewers were included in the mild group if they had a VF MD better than or equal to −6 dB and in the moderate-to-severe group with an MD worse than −6 dB. The mean and standard deviation of AUC of the best performing model in distinguishing between these three groups (healthy, mild functional loss, moderate-to-severe functional loss) was evaluated. The sensitivity of the model in identifying any, mild, and moderate-to-severe GON at varying levels of specificity was also computed to characterize model performance.

### Visualizing Model Decisions

Deep learning models have often been referred to as “black boxes” because it is difficult to know the process by which they make predictions. To help open this black box and understand what features are most important for model to identify GON in fundus images two strategies were employed: (1) qualitative evaluation of exemplar healthy and GON images along with model predictions and (2) occlusion testing to identify image regions with the greatest impact on model predictions. The qualitative assessment was performed by an expert grader (C.B.) who reviewed testing set images for which the model produced high confidence true predictions (true positives and negatives), high confidence false predictions (false positives and negatives), and low confidence predictions (borderline predictions). Examples of each of these predictions were selected to help illustrate model decisions and images that led to model errors.

The decision-making process of the model was also illustrated through the use of occlusion testing. This technique overlays a blank window on an image before applying the model to quantify the impact of each region on the model prediction. By passing this window over the entire image, the areas with the greatest impact on model predictions can be identified^42^. To identify ONH regions that had the greatest impact on model predictions, occlusion testing was performed on the entire set of testing images. For this analysis, only the right-eye orientation of each image was used and the average occlusion testing maps for healthy and GON eyes were computed.

## Results

Table 1 summarizes the clinical and demographic characteristics of the ONH photograph dataset for the healthy, mild, and moderate-to-severe glaucoma eyes considered in this work. The GON participants were older (64.0 vs. 55.3 years), had worse MDs (−4.1 vs −0.76 dB), and had a greater proportion of individual of African descent (45.5% vs. 28.0%) than healthy participants.

### Model Training and Selection

**Figure 3:**
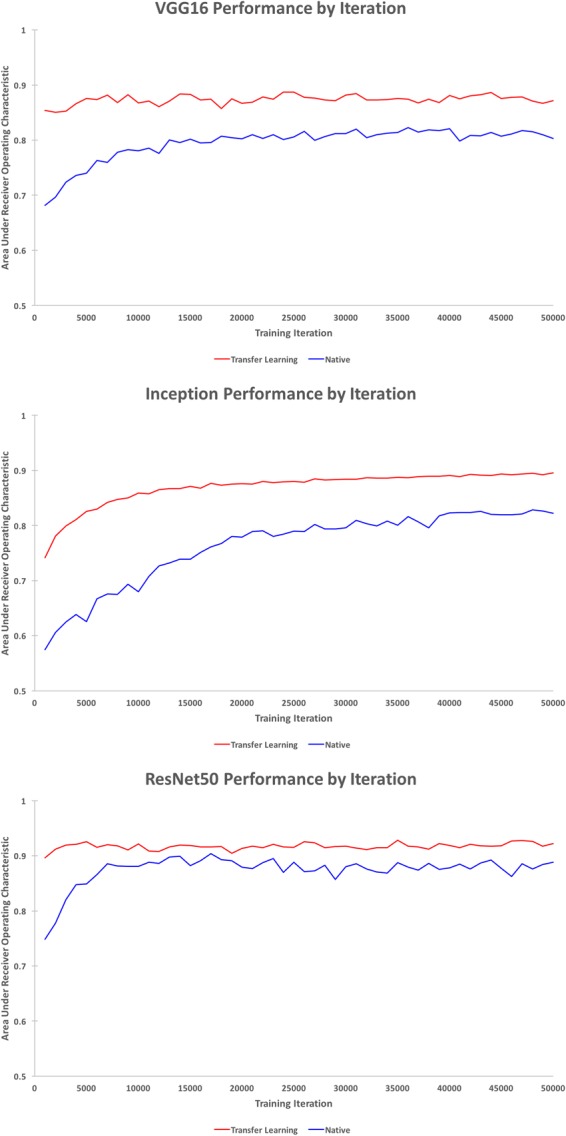
Model performance on the validation dataset by iteration for the first 50,000 training iterations for the VGG16 (top), Inception (middle), and ResNet50 (bottom) architectures.

Model performance was evaluated on the validation datasets after every 1,000 training iterations. Figure 3 illustrates model performance for the first 50,000 iterations. Regardless of architecture, transfer learning models had greater initial performance and tended to reach convergence in fewer iterations.

### Model Evaluation

**Figure 4:**
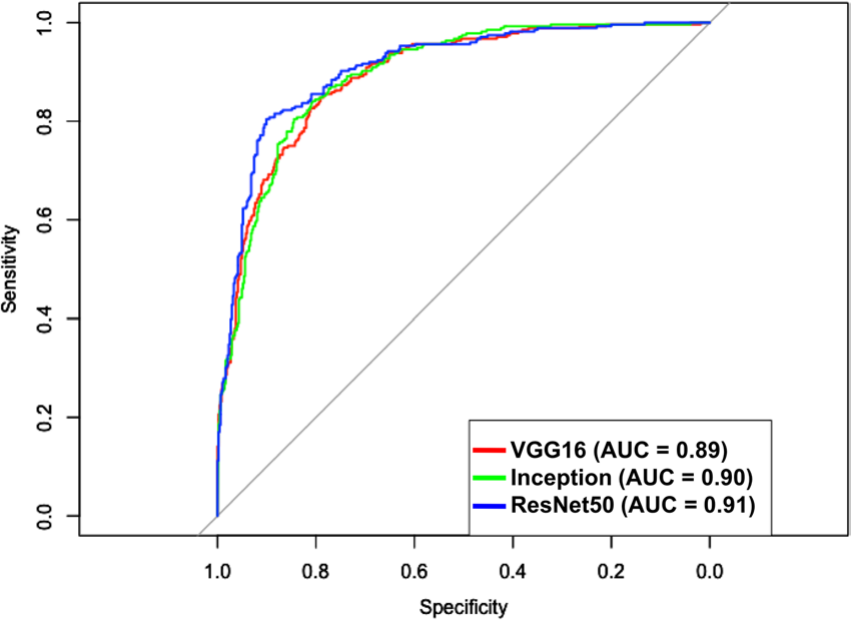
ROC curves in predicting glaucomatous optic neuropathy (GON) for each deep learning architecture initialized using transfer learning.

The native VGG16 model achieved an AUC of 0.83 (95% CI: 0.81–0.85) and the transfer learning VGG16 model achieved 0.89 (95% CI: 0.87–0.91) on the testing data. For the Inception model, native AUC was 0.83 (95% CI: 0.81–0.85) and transfer learning AUC was 0.90 (95% CI: 0.88–0.92). Finally, native ResNet50 AUC was 0.89 (95% CI: 0.87–0.91) and transfer learning ResNet50 achieved the highest overall AUC of 0.91 (95% CI: 0.90–0.93). This transfer learning ResNet model AUC was higher than all other models, significantly (p < 0.05) so in all cases except for the transfer learning inception model. See Fig. 4 for ROC curves and Table 3 for a summary of these results. In all cases, the transfer learning models outperformed their native counterparts on the testing dataset.

**Table 3:** GON detection accuracy of deep learning models on test dataset.

Model	AUC (95% CI)	P-value
VGG16		
Native	0.83 (0.81–0.85)	<0.001*
Transfer Learning	0.89 (0.87–0.91)	0.045*
Inception		
Native	0.83 (0.81–0.85)	<0.001*
Transfer Learning	0.90 (0.88–0.92)	0.11*
ResNet50		
Native	0.89 (0.87–0.91)	0.01*
Transfer Learning	0.91 (0.90–0.93)	—

*AUC significantly (p < 0.05) lower than the transfer learning ResNet50 model.

The best performing model (transfer learning ResNet50) was also evaluated based on its ability to distinguish healthy eyes from GON eyes with mild functional loss and GON eyes with moderate-to-severe functional loss. In identifying GON eyes with mild functional loss, the model achieved an AUC of 0.89 (95% CI: 0.88–0.90). In the case of GON eyes with moderate-to-severe loss, the model had an AUC of 0.97 (95% CI: 0.96–0.98). The transfer learning inception model achieved a sensitivity and specificity of 0.84 (95% CI: 0.83–0.85) and 0.83 (95% CI: 0.81–0.84) in identifying any GON, 0.82 (95% CI: 0.80–0.84) and 0.82 (95% CI: 0.80–0.83) in identifying mild GON, and 0.92 (95% CI: 0.89–0.94) and 0.93 (95% CI: 0.92–0.95) in identifying moderate-to-severe GON. Tables 4 and 5 summarize the full AUC, sensitivity, and specificity of the transfer learning Inception model.

**Table 4:** Accuracy of the best performing model (transfer learning ResNet50) in identifying any, mild, and moderate-to-severe glaucomatous optic neuropathy shown along with 95% CI.

Metric	GON Level		
	Any	Mild	Moderate-to-Severe
AUC	0.91 (0.90–0.91)	0.89 (0.88–0.90)	0.97 (0.96–0.98)
Sensitivity	0.84 (0.83–0.85)	0.82 (0.80–0.84)	0.92 (0.89–0.94)
Specificity	0.83 (0.81–0.84)	0.82 (0.80–0.83)	0.93 (0.92–0.95)

**Table 5:** Sensitivity of the best performing model (transfer learning ResNet50) in identifying any, mild, and, moderate-to-severe GON at varying levels of specificity.

Specificity	Sensitivity by Disease Severity		
	Any	Mild	Moderate-to-Severe
0.80	0.85 (0.83–0.87)	0.83 (0.80–0.85)	0.96 (0.94–0.98)
0.85	0.80 (0.78–0.83)	0.77 (0.74–0.80)	0.95 (0.92–0.98)
0.90	0.74 (0.71–0.76)	0.69 (0.65–0.72)	0.93 (0.89–0.96)
0.95	0.62 (0.59–0.65)	0.55 (0.51–0.59)	0.88 (0.85–0.91)

### Visualizing Model Decisions

**Figure 5:**
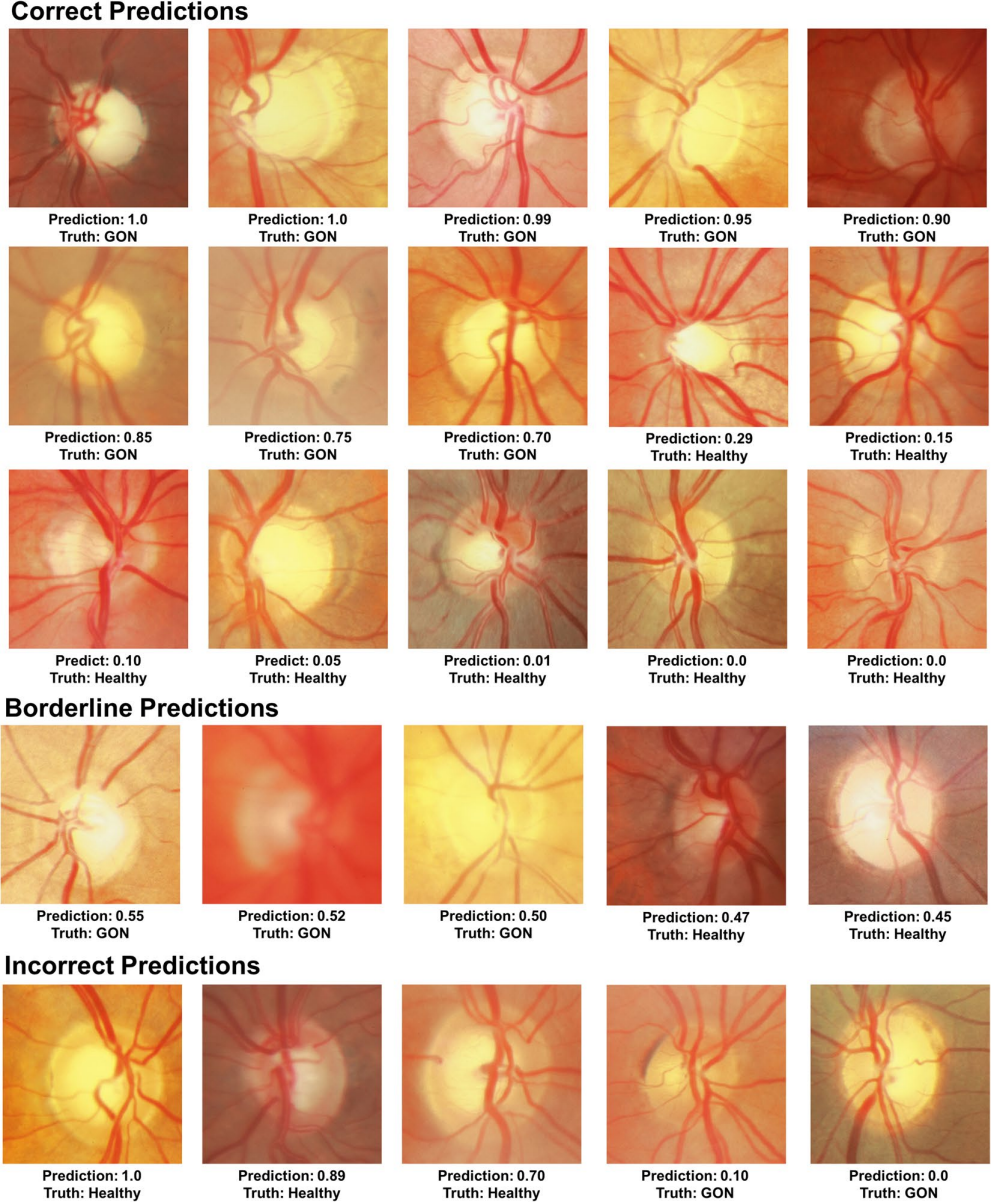
Predictions of the best performing model compared to expert truth. Examples of correct, incorrect, and borderline predictions are shown along with the expert truth and model glaucomatous optic neuropathy (GON) prediction probabilities (0 = healthy, 1 = GON).

Figure 5 provides case examples of model predictions and expert-based truth. A sampling of correct, incorrect, and borderline examples is shown along with the model GON predictions and truth. Among the correct GON predictions, hallmarks of glaucoma are visible (localized and diffuse rim thinning) and absent in the correctly identified healthy examples. The borderline cases suffer from more image quality issues (blurring, poor contrast) than the other examples. In the incorrect cases, some healthy examples predicted as GON (false positives) exhibited some characteristics typically associated with glaucoma such large vertical cup-to-disc ratios on the order of 0.8.

**Figure 6:**
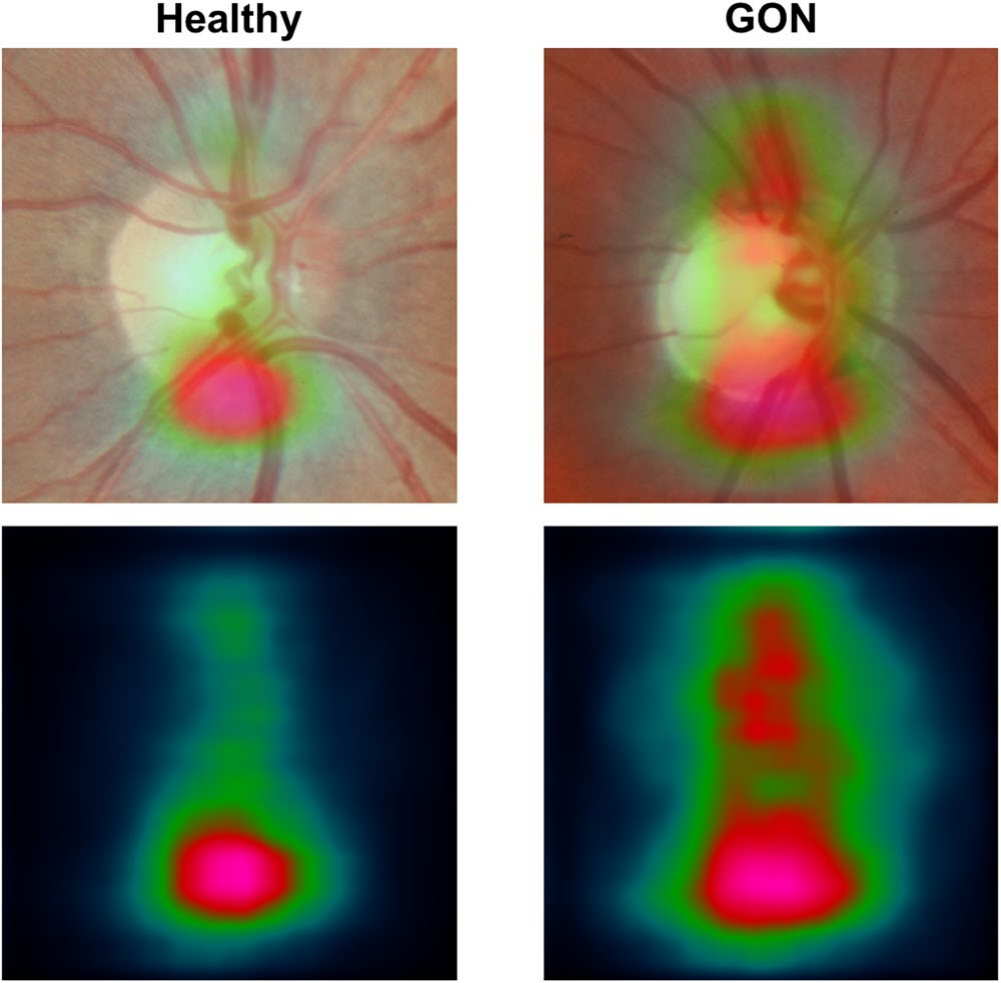
Mean occlusion testing maps showing most significant regions for distinguishing healthy and glaucomatous optic neuropathy (GON) images. In these images, bright pink regions indicate a large impact on model predictions while dark blue regions indicate a very limited impact on predictions. The maps were generated by averaging occlusion testing maps of the healthy and GON testing images in right eye orientation. The heat maps (bottom) are shown overlaid on a representative healthy and GON eye (top).

Figure 6 shows the results of occlusion testing to identify ONH areas that had the greatest impact on model decisions. Average occlusion testing maps are shown for each group to illustrate regions most important to identifying healthy and GON images. For both healthy and GON eyes, the neuroretinal rim areas were identified as most important and the periphery contributed comparatively little to model decisions. Specifically, a region of the inferior rim was identified as the most important in the average occlusion maps. In GON eyes (and healthy eyes, to a lesser extent), a secondary region along the superior rim and arcuate area was also identified as important.

## Discussion

Our results suggest that deep learning methodologies have high diagnostic accuracy for identifying fundus photographs with glaucomatous damage to the ONH in a racially and ethnically diverse population. The best performing model was the transfer learning ResNet architecture and achieved an AUC of 0.91 in identifying GON from fundus photographs, outperforming previously published accuracies of automated systems for identifying GON in fundus images. These included systems based on traditional image processing and machine learning classifiers as well as those that used deep learning approaches (AUCs in range of 0.80–0.89)^43–45^. The model had even higher performance (AUC of 0.97, sensitivity of 90% at 93% specificity) in identifying GON in eyes with moderate-to-severe functional damage.

The accuracy of this approach may help make automated screening programs for glaucoma based on ONH photographs a viable option to improve disease detection in addition to providing decision-making support for eye care providers. Previous work has shown that limited sensitivity and specificity of tests reduce the feasibility of screening programs given low overall disease prevalence^8,46,47^. The model described here achieved a sensitivity of 0.92 and specificity of 0.93 in identifying moderate-to-severe GON. This suggests that it may be able to accurately identify GON in specific screening situations while reducing the burden of false positives compared to other glaucoma-related measurements. Automated (rather than human) review of fundus photographs would also help reduce costs and aid in the implementation of large-scale screening programs by providing quick, objective and consistent image assessment. This deep learning model for glaucoma detection using ONH photographs could also be combined with deep learning-based assessment of fundus photography for referable diabetic retinopathy that has recently been approved by the FDA for use in primary care clinics.

Deep learning based review of fundus images could also be useful in glaucoma management as part of clinical decision support systems through incorporation into fundus cameras, electronic medical records, or PACS systems. The quantitative GON probabilities provided by these models serve as a confidence score regarding the GON prediction – a borderline score near 0.5 may suggest additional scrutiny compared to a score of 0.0 or 1.0. Application of these models can also provide guidance to human reviewers about what specific image regions contributed to the model prediction (e.g. through occlusion testing). Clinicians can incorporate model predictions and guidance along with all other patient-specific data for patient management decisions.

To help understand and visualize model decisions, a sample of the correct, incorrect, and borderline testing set examples were reviewed by an expert grader. Based on this additional review, the model does seem to identify glaucoma-related ONH features (e.g. rim thinning). However, image quality issues such as blurring or low contrast can cause model errors and low confidence (~0.50) predictions. For additional insight into model decisions, an occlusion testing procedure was performed. This technique identified areas of input images that had the greatest impact on model output. To identify the regions that were most important across the entire dataset, average occlusion maps were computed for healthy and GON images (Fig. 6). These results show that, for this dataset, the model identified inferior and superior regions of the neuroretinal rim as the most important part of the posterior ocular fundus for distinguishing between GON and healthy eyes. These regions correspond to a longstanding rule-of-thumb in ONH evaluation that glaucoma damage often occurs first in the inferior region followed by the superior regions^48^. Based on occlusion testing, part of the model determination may be based on early changes or thinning of the thickest rim regions (inferior and superior). These hot spots both conform with areas of the disc and adjacent retina previously found to distinguish between GON and healthy eyes. This suggests that (in part) our model evaluates fundus photographs in a way similar to clinicians.

Li *et al*. recently published their results in training a deep learning model (using the Inception architecture) for identifying GON in fundus images^49^. Their deep learning model achieved an AUC of 0.986 in distinguishing healthy from GON eyes, similar to our AUC of 0.97 for moderate to severe glaucoma. Several differences between the datasets may help account for this difference. First, the dataset used in Li *et al*. (LabelMe, http://www.labelme.org) contained a greater number of images (~39,000 vs. ~7,000 fundus images) and eyes (~39,000 vs. ~4,000 eyes). Because of the high data requirements of deep learning models, the addition of thousands of fundus images would help improve performance substantially. Moreover, while the decision healthy vs. GON was left up to grader judgement in this work, Li *et al*. used stricter criteria for defining GON. Images were required to display at least one of the following to be labeled as GON: cup-to-disc ≥0.70, localized notching with rim width ≤0.1 disc diameter, visible nerve fiber layer defect, or disc hemorrhage. The required presence of one or more of these features likely meant that the GON images in Li *et al*. represented more advanced disease than many GON images in the current study. Comparing our performance in identifying moderate-to-severe GON, the models achieved similar performance (0.97 vs. 0.986 AUC). In addition, their dataset was collected from a more racially homogenous population. The LabelMe dataset used in Li *et al*. was collected from an almost exclusively Chinese population, while the data considered here was collected from a more geographically and racially diverse population of individual of European descent (~60%) and African descent (~35%) from California, Alabama and New York. There are important differences in ONH appearance and structure across different populations including differences in disc size, cup and rim size, and cup-to-disc ratio^50^. The heterogeneity of our dataset meant that our model needed to learn GON-related features in different racial groups. Stratifying the test set by race, suggests that the deep learning model provides similar accuracy in individuals of European descent and individuals of African descent with AUCs of 0.91 (0.89–0.92) and 0.90 (0.89–0.92 (p = 0.83), respectively. The diverse population of our training, validation, and testing datasets may have resulted in a more generalizable model. However, these results are limited in that only two groups (European and African descent) comprised ~95% of the participants. Evaluation and training on independent datasets with greater representation of additional populations will help quantify and improve model generalizability.

Comparing the results of different deep learning architectures (VGG16 vs. Inception vs. ResNet50) and strategies (native vs. transfer learning) may help provide guidance for future development of models to identify GON. The performance of the ResNet50 architecture was significantly better than that of the VGG16 and Inception architectures. These results are consistent with the relative performance of these architectures on general image classification tasks. ResNet incorporates residual layers to help aid the model achieve convergence during training of very deep networks such as these^51^. These residual layers seemed to aid the model in learning GON-related image features and resulted in significantly higher performance.

For all deep learning architectures, transfer learning increased performance in detecting GON and decreased the training time needed for model convergence. A possible reason that transfer learning helps the model learn faster with less data is that it mimics the way our visual system develops. The visual system develops circuits to identify simple features such (e.g. edges and orientation), combines these features to process more complex scenes (e.g. extracting and identifying objects), and associates this complex set of features with other knowledge regarding the scene. By the time a clinician learns to interpret medical images, they already have vast experience in interpreting a wide variety of visual scenes. Initializing models via transfer learning is an important approach that should be considered whenever training a CNN to perform a new task, especially when limited data is a concern.

There are some limitations to this study. The ground truth used here was derived from the generally accepted gold standard of subjective assessment of stereoscopic fundus photography by at least 2 graders certified by the UCSD Optic Disc Reading Center with adjudication by a third experienced grader in cases of disagreement. Because the images were collected and reviewed over the course of ~15 years, the ground truth was not generated by a single pair of graders and a single adjudicator. This means that model classifications cannot be compared to 2 specific graders to determine agreement. Rather, our results were compared to the ground truth based on the final grade. For our dataset, two graders agreed in the assessment of an image in approximately 77% of cases and adjudication was required in approximately 23% of cases. This is comparable to previously published levels of agreement between graders and in ground truth data used in training automated systems^52,53^.

For our analysis, stereo pairs were separated and treated as separate, non-stereo images during classification. In many circumstances, however, stereoscopic photographs are not available for GON evaluation and are generally not used in screening situations. Thus, while including stereoscopic information may eventually enhance performance, separating stereo pairs for our analysis mimics situations in which stereo information is not available and increases the generalizability of our results to non-mydriatic cameras used in population-based and primary care based screening programs^6^. A limitation of the current results, however, is that the effect of specific camera type/model on performance could not be quantified. For this retrospective dataset, the original film-based camera model and settings were not available. The digitization of the film photographs, however, was standardized and consistent. Future work should include training and evaluation of models on images collected using multiple, known cameras. This would aid clinicians in selecting deep learning models most appropriate for their imaging instruments.

Additionally, the ONH region was extracted from the images and used as input to the model. This was done to ensure that the model was exposed to a fairly consistent, ONH-centered view. During expert review for this study, GON grades were assigned using the information provided by stereo photos that included areas peripheral to the disc with visual information on the retinal nerve fiber layer available for some photographs. For example, *post-hoc* assessment of full stereo photographs from healthy eyes that were incorrectly predicted as GON revealed healthy superior and inferior arcuate RNFL bundles outside the region of the cropped ONH image (Fig. 5). Considering only the cropped ONH region, the large cup-to-disc ratios and possible rim thinning likely led to the incorrect model prediction. In other cases, however, healthy eyes with large discs or cups were correctly classified by the model even without the additional context provided by the full stereo photographs. Future work should investigate the effect of the ONH region size, resolution, and incorporation of stereo information on model performance. Incorporating more of the periphery and stereo information may help to improve identification of glaucomatous damage^54^.

In addition, the dataset under consideration included multiple images captured over several years from most participants. This longitudinal component contains important information and clinicians regularly evaluate fundus photographs in comparison to a baseline photograph to determine the extent (or rate) of glaucomatous damage. For the current study, the longitudinal aspect of the data was not considered and the entire dataset was used train models that considered each image individually. In addition to cross sectional data, there is potential for the analysis of longitudinal data to detect progression using deep learning approaches. Although standard deep learning CNN architectures are not designed to work on longitudinal data, there are recurrent CNN variants that consider a sequence of images^55^. These recurrent networks include a temporal component so that information from multiple images captured sequentially can be combined to produce a single prediction. Using the dataset described here, future work could include training models that use longitudinal images to increase the confidence of GON predictions, estimate the incidence of glaucoma progression, or predict the extent and type of damage at a future time point.

In summary, the best performing deep learning model had expert-level accuracy (AUC = 0.91) in identifying any GON and even higher accuracy (AUC = 0.97) in identifying GON with moderate-to-severe functional damage. Given the increasing burden of glaucoma on the healthcare system as our population ages and the proliferation of ophthalmic image devices, automated image analysis methods will serve an important role in decision support systems for patient management and in population- and primary care-based screening approaches for glaucoma detection. The results presented here suggest that deep learning based review of ONH images is a viable option to help fill this need.
